# An iTRAQ-based proteomic analysis reveals dysregulation of neocortical synaptopodin in Lewy body dementias

**DOI:** 10.1186/s13041-017-0316-9

**Published:** 2017-08-11

**Authors:** Arnab Datta, Yuek Ling Chai, Jing Min Tan, Jasinda H. Lee, Paul T. Francis, Christopher P. Chen, Siu Kwan Sze, Mitchell K. P. Lai

**Affiliations:** 1 Department of Pharmacology, Yong Loo Lin School of Medicine, National University of Singapore, Unit 09-01, Centre for Translational Medicine (MD6), 14 Medical Drive, Kent Ridge, Singapore 117599, Singapore; 2 Epithelial Systems Biology Laboratory, National Heart, Lung, and Blood Institute, Building 10 Room 6N318, Bethesda, MD 20814 USA; 3 Wolfson Centre for Age-related Diseases, King’s College London, Guy’s Campus, St Thomas Street, London SE1 1UL, UK; 4 School of Biological Sciences, Nanyang Technological University, Singapore 637551, Singapore

**Keywords:** Lewy body dementias, Dementia with Lewy bodies, Parkinson’s disease dementia, iTRAQ, Clinical proteomics, β-amyloid, Aβ42, Synaptopodin

## Abstract

Lewy body dementias are the second most common cause of neurodegenerative dementia in the elderly after Alzheimer’s disease (AD). The two clinical subgroups of Lewy body dementias, namely, dementia with Lewy bodies (DLB) and Parkinson’s disease dementia (PDD), are differentiated by the chronology of cognitive symptoms relative to parkinsonism. At present, there remains a debate on whether DLB and PDD are separate disease entities, or fall within the same spectrum of Lewy body dementias. In this study, we compared the detergent-soluble proteome via an 8-plex isobaric tag for relative and absolute quantitation (iTRAQ) analysis of pooled lysates from the prefrontal cortex (BA9) of DLB (*n* = 19) and PDD (*n* = 21) patients matched *a priori* for amyloid (total Aβ42) burden, semi-quantitative scores for Lewy bodies and neurofibrillary tangles together with age-matched control (*n* = 21) subjects. A total of 1914 proteins were confidently identified by iTRAQ (false discovery rate = 0%). None of the proteins showed a significant yet opposite regulation in between DLB and PDD when compared to aged controls in the proteomic data set as well as following immunoblot analysis of the pooled and individual lysates involving all 61 subjects. The postsynaptic protein, synaptopodin (SYNPO) was significantly down-regulated in both DLB and PDD subgroups, suggesting a defective synaptic transmission in the demented patients. In conclusion, the largely similar proteome of DLB and PDD matched for amyloid burden suggests that variations in concomitant AD-related pathology, abnormal post-translational modifications or protein-protein interactions, defective intracellular trafficking or misfolding of proteins could play a part in driving the clinically observed differences between these two subgroups of Lewy body dementias. This further indicates that amyloid-targeting therapeutic strategies may show different efficacies in DLB versus PDD.

**Electronic supplementary material:**

The online version of this article (doi:10.1186/s13041-017-0316-9) contains supplementary material, which is available to authorized users.

## Introduction

Lewy body dementias are increasingly being recognized as a common cause of old-age dementia, being the third most prevalent (15–20%) after Alzheimer’s disease (AD) and vascular dementia (VaD) [1]. They are characterized by the presence of cortical α-synuclein-positive Lewy bodies (LBs) and Parkinsonian extrapyramidal motor symptoms. The two main clinical subtypes of Lewy body dementias; dementia with Lewy bodies (DLB) and Parkinson’s disease dementia (PDD) are distinguished by the temporal relationship between the onset of dementia and motor symptoms. Therefore, the onset of dementia preceding, or occurring within one year of motor symptoms are diagnosed as DLB, while dementia occurring one year or more after motor symptoms should be termed as PDD. This arbitrarily defined ‘one-year rule’ resulted in an ongoing debate on whether DLB and PDD are “different points on a common spectrum of Lewy body disease” or are distinct clinical syndromes [2–4]. DLB and PDD share clinical features of executive impairment and fluctuating attention with relatively mild memory impairment [5–7], although minor differences may exist in specific domain subscores [8]. DLB and PDD also manifest similar severity of motor symptoms [9, 10], comparable Lewy body burden in most cortical regions [11], and cortical neurochemical alterations of cholinergic and dopaminergic deficits as well as losses of nicotinic and glutamatergic GluA receptors [12–14]. More recent neurochemical studies also reported deficits in zinc transport and postsynaptic markers in both DLB and PDD [15]. On the other hand, DLB has variable, but generally higher, amyloid plaque load compared to PDD [11, 16, 17] as well as more severe executive impairment and visual hallucinations [3]. In this regard, a systematic approach to studying changes in proteomic landscape may uncover other similarities and differences between DLB and PDD.

Tissue-based quantitative clinical proteomics has emerged as an unbiased mechanistic and discovery tool to study various neurological disorders. We had pioneered the successful coupling of isobaric labeling with two dimensional-liquid chromatography-tandem mass spectrometry (iTRAQ-2D-LC-MS/MS) to propose potential therapeutic targets and biomarkers in ischemic stroke and VaD [18–20]. Although a similar clinical proteomic approach has been used to study Parkinson’s disease (PD), few studies have focused on Lewy body dementias exclusively [21]. Incidentally, two of them that used an iTRAQ experiment, included either PDD or DLB as one of the groups and no direct comparison was made between these two subtypes of Lewy body dementias. Further, they have used cerebrospinal fluid (CSF) as a starting sample with an objective to propose candidate biomarkers for further validation [22, 23]. We could find only one study that compared brain tissues of a familial Parkinsonism-dementia complex and DLB on an iTRAQ platform [24], while studies directly comparing PDD and DLB have not been reported. Clearly, tissue-based quantitative clinical proteomics remained underutilized despite its potential to offer a comprehensive insight into the underlying mechanisms of Lewy body dementia.

Here, using pooled neocortical lysates from a well-characterized cohort of PDD, DLB and matched control subjects, we extend the application of the iTRAQ-guided discovery approach to determine whether these two clinical subtypes of Lewy body dementias have a divergent molecular signature or share similar aberrant pathways with varying extent of deregulation. The detergent-soluble proteomic dataset was filtered with rigorous selection criteria to shortlist differentially expressed proteins for a two-step immunoblot experiment on pooled and individual samples. Proteome-wide comparison of DLB and PDD preselected for comparable total amyloid β-42 (Aβ42) protein and amyloid plaque load revealed a grossly similar pattern of protein expression with a variation in the magnitude of deregulation between these two disorders, indicating an overlapping pathology between DLB and PDD.

## Results

The study was divided into three phases; phase –I: Pre- selection of subjects, phase –II: discovery proteomics and phase – III: post-proteomic data validation.

### Phase –I: Sample matching using total Aβ42 levels

**Fig. 1:**
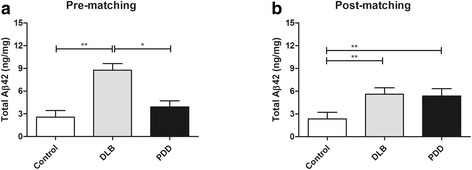
Bar charts showing concentrations of total Aβ42 in BA9. **a** the complete cohort containing 109 subjects and **b** the 61 subjects (21 Controls, 19 dementia with Lewy Bodies [DLB], 21 Parkinson’s disease dementia [PDD]) selected for the current study. Data were presented as mean ± SEM of total Aβ42 (ng/mg brain protein). Significant difference (Kruskal Wallis ANOVA followed by *post-hoc* Dunn’s tests) *p < 0.05 or **p < 0.01

Brain tissue was available from the BA9 area of frontal cortex of 109 subjects (23 Controls, 52 DLB and 34 PDD). We determined the concentrations of Aβ42 in BA9 area by ELISA, which showed significantly higher levels in DLB group compared to the control and PDD groups (Fig. 1a), in corroboration with previous studies showing increased Aβ42 load in DLB [25–27]. To study proteomic changes resulting from processes independent from those related to AD, we selected a total of 40 Lewy body dementia (19 DLB and 21 PDD) patients matched a priori for total Aβ42 concentration, together with 21 control subjects. Other clinical or pathological features were not considered in the selection of the Lewy body dementia subjects. Thus, the mean levels of total Aβ42 in DLB and PDD did not differ significantly, although the DLB group still had significantly higher total Aβ42 compared to the Control group (Fig. 1b).

### Demographic and disease variables of participants

**Fig. 2:**
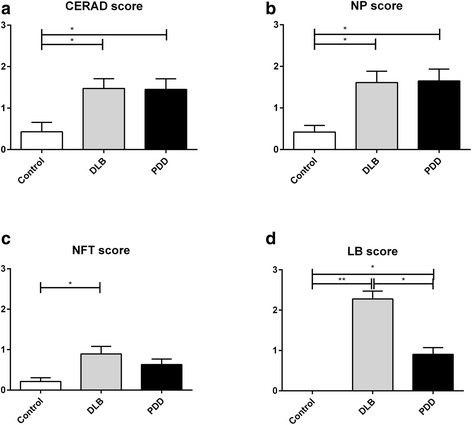
Bar charts showing the neuropathological scores (**a** CERAD, **b** neuritic plaque [NP], **c** neurofibrillary tangle [NFT] and **d** Lewy Body [LB]) in the subjects (21 Controls, 19 DLB, 21 PDD), selected for iTRAQ experiment. Data were presented as mean ± SEM. Significant difference (Kruskal Wallis ANOVA followed by *post-hoc* Dunn’s tests) *p < 0.05 or **p < 0.01

Demographic and disease variables of the 61 selected subjects are summarized in Table 1. Age, gender (as % male), post-mortem interval and tissue pH (as a measure of agonal status [28]) were matched among control, PDD and DLB (one-way analyses of variance, (ANOVA) with Bonferroni *post-hoc* tests, *p* > 0.05). PDD patients had longer duration of Parkinson’s disease symptoms, while DLB showed longer duration of dementia (Student’s *t*-tests, *p* < 0.05). This observation is consistent with the respective predominant clinical features of PDD (parkinsonism) and DLB (dementia) over the course of the disease, and indicated clear differentiation of clinical phenotypes between the disease groups. Lastly, majority of the controls had Braak staging ≤ II and none had Braak staging > IV, confirming the presence of only mild age-related pathological changes (Table 1). Figure 2 shows that a priori matching of amyloid plaque load in DLB and PDD resulted, as expected, in comparable Consortium to Establish a Registry for Alzheimer’s Disease (CERAD) scores and neuritic plaques (NP) scores. Neurofibrillary tangles (NFT) scores also did not differ between DLB and PDD (Fig. 2c). This confirms the matching of AD-related pathology between DLB and PDD in our study. However, LB scores remained higher in DLB compared to PDD (Fig. 2d), in line with previous observations [27].

**Table 1:** Demographic, neurochemical and disease variables of subjects included in the study

Demographics		Control	DLB	PDD
Number of cases		21	19	21
Age at death (mean yrs. ± SD)		81.7 ± 6.5	80.7 ± 6.2	78.8 ± 6.1
Sex (Male,%)		13 (61.9)	10 (52.6)	11 (52.3)
Post-mortem interval (mean hrs ± SD)		35.2 ± 22.7	31.8 ± 18.6	33.7 ± 16.1
Tissue pH		6.5 ± 0.3	6.4 ± 0.5	6.5 ± 0.3
Braak Staging (n)	0-II	14	2	13
	III-IV	1	13	6
	V-VI	0	4	2
	NA	6	0	0
CERAD Score (n)	None	10	3	5
	Sparse	3	8	6
	Moderate	0	4	4
	Frequent	1	4	5
	NA	7	0	1
Lewy Body Score (n)	None	19	0	7
	Sparse	0	4	9
	Moderate	0	5	5
	Severe	0	9	0
	NA	2	1	0

*Abbreviations: DLB,* dementia with Lewy body; *PDD,* Parkinson’s disease dementia; *n,* number; *NA,* not available

### Phase II: Discovery proteomics

To understand the global proteomic changes that occurred in the BA9 area of the prefrontal cortex of Lewy body dementias, we pooled equal amount of protein from selected subjects group-wise to generate the pooled lysates for PDD (*n* = 21), DLB (*n* = 19), and control (*n* = 21) groups. Further, equal amounts of protein from all 40 dementia (DLB and PDD) patients were pooled to generate the ‘Dementias’ group. Sample pooling strategy has been used widely to reduce the effect of biological variation while dealing with clinical samples [19, 24, 29]. The labeling scheme for 8-plex iTRAQ experiment was as follows; control (label: 113, 116), DLB (label: 114, 117), PDD (label: 115, 118), Dementias (label: 119) and pooled groups (label: 121), where all samples across groups were combined.

### Quality control of the iTRAQ dataset

To minimize the false positive identification of proteins, we used a strict cutoff of unused ProtScore ≥ 2 as the qualification criteria, which corresponds to a peptide confidence level of > 99%. With this criterion, 1914 proteins were identified from the iTRAQ experiment with a false discovery rates (FDR) of 0% from the combined data set containing three technical replicates (Additional file 1: Table S1). The average number of unique peptides (having a confidence level of ≥ 95%) detected per protein was 6.8. More than 29.1% of the proteins (557 proteins) had ≥ 5 unique peptides, while around 2.1% of proteins (40 proteins) were identified with ≥ 50 peptide. The average %coverage for the combined data set was 11.1%, whereas around 30.4% of the proteins (581 proteins) had % coverage more than the average level.

### Estimation of threshold for confidently defining perturbed proteins

We determined the cutoff for up- or down-regulation based on the label-specific experimental variation between two replicates for the three experimental groups (control: 113, 116; DLB: 114, 117; PDD, 115, 118) [19, 30]. It should be noted here that variations may arise from handling and transfer of the samples during gel loading, in-gel digestion, extraction of digested peptides or during isobaric labeling. A total of 1988 proteins having ratios were included for this analysis. Ideally, the ratios between the experimental replicates (116/113, control; 117/114, DLB; 118/115, PDD) should be equal to one. The frequency distribution of the fold of deviation from one was measured and plotted for the three above-mentioned ratios (Additional file 2: Figure S1). The average experimental variation was 1.11-fold. The experimental variation was ≤1.3 fold for around 93%, 96% and 94% of the ratios for control, DLB and PDD groups. Based on this, the regulation cut-off was set 1.3-fold; ratio > 1.30 and < 0.77 was considered as up- or down-regulated.

As mentioned above, using duplicate iTRAQ labels for control, DLB and PDD groups, four ratios were calculated each for DLB (114/113, 117/113, 114/116, 117/116) and PDD (115/113, 118/113, 115/116, 118/116) group with respect to control, while Dementias group was represented by two ratios (119/113, 119/116). Overall, the dataset contained ten different ratios when all combinations of replicates were considered. Next, the dataset was filtered based on the following criteria. (a) The ratios representing DLB, PDD and Dementias were sorted using a *p*-value cut-off of 0.05 (*p* < 0.05) and a regulation threshold of 1.3-fold to obtain the list of significantly perturbed proteins. Thirty-eight proteins with a significant *p*-value for at least one of these 10 calculated ratios and a magnitude beyond 1.3-fold were short-listed. (b) Finally, geometric mean was calculated from the ratios corresponding to DLB, PDD and Dementias and filtered with the cut-off of 1.3-fold to shortlist seven proteins (Table 2).

**Table 2:** Final list of proteins filtered from the complete list of confidently identified proteins^*^

ID	N	Unused	%Cov(95)	Name	Peptides (95%)	GM (DLB/Control)	%CV	GM (PDD/Control)	%CV	GM (Dementias/Control)	%CV
P04406	20	59.4	61.5	G3P_HUMAN Glyceraldehyde-3-phosphate dehydrogenase GN = GAPDH	67	1.08	6.8	1.09	5.0	1.03	6.5
P08670	56	38.0	43.6	**VIME_HUMAN Vimentin GN = VIM**	26	1.24	8.7	0.86	6.8	1.03	8.8
P11137	81	29.9	11.9	MAP2_HUMAN Microtubule-associated protein 2 GN = MAP2	16	0.81	6.8	**0.72**	7.1	**0.76**	8.6
P13591	119	23.4	16.1	**NCAM1_HUMAN Neural cell adhesion molecule 1 GN = NCAM1**	17	1.05	5.7	0.99	7.3	0.98	7.0
P20336	167	19.7	52.7	RAB3A_HUMAN Ras-related protein Rab-3A GN = RAB3A	18	0.98	15.5	0.95	14.1	0.97	15.8
P60880	184	18.6	50.0	SNP25_HUMAN Synaptosomal-associated protein 25 GN = SNAP25	18	0.97	3.1	0.95	2.9	1.00	2.3
Q5HY54	186	18.3	3.2	Q5HY54_HUMAN Filamin A, alpha (Actin binding protein 280) GN = FLNA	7	0.96	2.2	**0.75**	2.1	0.83	0.6
A8K0Y4	241	15.3	39.4	A8K0Y4_HUMAN cDNA FLJ75013 GN = GAP43	12	1.11	11.0	1.09	11.5	1.08	12.2
P09211	242	15.3	48.6	**GSTP1_HUMAN Glutathione S-transferase P GN = GSTP1**	10	1.15	7.0	1.19	7.7	**1.30**	7.7
P60201	293	13.6	19.9	**MYPR_HUMAN Myelin proteolipid protein GN = PLP1**	24	1.18	2.6	1.24	2.6	1.22	0.3
Q4W5L2	359	11.7	65.7	Q4W5L2_HUMAN Putative uncharacterized protein SNCA (Fragment) GN = SNCA	6	0.99	1.6	1.08	1.6	1.08	0.4
P00441	369	11.5	69.5	SODC_HUMAN Superoxide dismutase [Cu-Zn] GN = SOD1	7	1.12	12.9	1.07	15.2	1.20	15.2
P08247	403	10.9	17.9	SYPH_HUMAN Synaptophysin GN = SYP	12	1.07	3.0	1.04	4.0	1.12	0.7
P12111	413	10.6	1.4	CO6A3_HUMAN Collagen alpha-3(VI) chain GN = COL6A3	4	1.02	3.1	**0.58**	5.7	0.78	3.4
Q9GZV7	419	10.4	17.4	HPLN2_HUMAN Hyaluronan and proteoglycan link protein 2 GN = HAPLN2	6	**1.32**	4.6	**1.33**	4.3	**1.39**	4.8
Q9BSJ8	475	9.4	4.6	ESYT1_HUMAN Extended synaptotagmin-1 GN = ESYT1	4	1.04	5.1	1.02	5.0	1.02	5.4
A7MD96	483	9.3	8.2	**A7MD96_HUMAN SYNPO protein (Fragment) GN = SYNPO**	4	**0.62**	5.8	**0.59**	3.0	**0.63**	1.7
P51674	568	8.0	13.0	GPM6A_HUMAN Neuronal membrane glycoprotein M6-a GN = GPM6A	5	1.05	12.5	1.18	12.0	**1.34**	13.4
P05067	675	6.6	5.5	A4_HUMAN Amyloid beta A4 protein GN = APP	3	1.03	6.8	1.02	8.5	0.95	7.9
Q8N9I0	1154	3.8	10.0	SYT2_HUMAN Synaptotagmin-2 GN = SYT2	4	1.21	11.7	1.22	15.3	1.17	14.6
O43581	1180	3.7	2.7	SYT7_HUMAN Synaptotagmin-7 GN = SYT7	2	0.98	8.1	0.98	7.9	0.91	10.1
Q8IV01	1198	3.5	5.7	SYT12_HUMAN Synaptotagmin-12 GN = SYT12	2	0.77	7.6	0.86	0.4	0.80	0.8

*The list contains 22 proteins with the quantization ratios for DLB, PDD and Dementias group with respect to control. Each group except ‘Dementias’ (label: 119) was labeled with two iTRAQ labels (control: 113, 116; DLB: 114, 117; PDD, 115, 118). Geometric means (GM) and % co-efficient of variation (% CV) were calculated from four ratios for DLB, PDD and from two ratios for the ‘Dementias’ group. The ratios in **bold** have qualified through the rigorous filtration criteria. The extent and trend of deregulation was largely similar for majority of the proteins between DLB and PDD. VIM was the only protein exhibiting opposite regulation between DLB and PDD. The protein names in **bold** were validated by immunoblot analysis of pooled lysates. ID, Uniprot identification; %Cov (95),%Coverage(95); GN, gene symbol

Considering a situation where the use of too stringent parameters during dataset filtration may result in the exclusion of an elusive marker protein between DLB and PDD, we manually curated the iTRAQ dataset to select additional candidate proteins from the extended list. For example, vimentin (VIM) was included as it showed an opposite trend of regulation between DLB (upward trend) and PDD (downward trend) without crossing the stipulated threshold of regulation (i.e. 1.3-fold). Neural cell adhesion molecule 1 (NCAM1) and superoxide dismutase 1 (SOD1) were included as a negative control as they were significantly elevated in BA21 area of subjects with VaD in one of our recently concluded study [19]. Conversely, SOD1 displayed a uniform reduction in the infarcted tissue of stroke patients when sampled from three different locations of the brain [18]. Overall, the final list contained twenty-two proteins (Table 2).

### Phase III: Post-proteomic immunoblotting

#### iTRAQ data validation

**Fig. 3:**
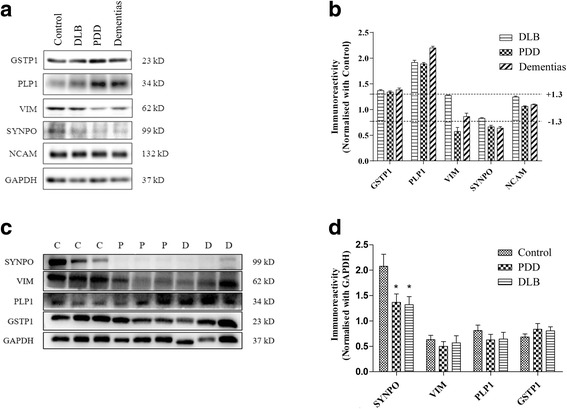
Two-step immunoblot analysis of selected proteins. **a** Representative immunoblots from the pooled lysates showing the protein levels of Control, DLB, PDD and the combined ‘Dementias’ group, with GAPDH used as the loading control. **b** Bar chart of immunoreactivities (mean ± SEM in arbitrary units) for comparing protein expression levels (with mean control values set at 1.0). Dotted lines were drawn at ± 1.3-fold to indicate the threshold of deregulation as determined from the iTRAQ experiment. The data shows upward trends for GSTP1, PLP1 and downward trends for SYNPO and VIM while NCAM did not exhibit any change between different groups. **c** Representative immunoblots of selected proteins using individual subjects. Control (C), PDD (P) and DLB (D) subjects selected randomly from each group for different proteins. GAPDH was used as the loading control. **d** Bar chart of normalized immuno-reactivities (mean ± SEM in arbitrary units) of the proteins of interest was calculated from all subjects (21 Controls, 19 DLB, 21 PDD) selected for the iTRAQ experiment. Significant difference (one-way ANOVA followed by post-hoc Bonferroni tests) *p < 0.05

We selected seven proteins (VIM, synaptopodin (SYNPO), glutathione S-transferase P (GSTP1), NCAM1, collagen alpha-3(VI) chain (COL6A3), filamin A, alpha (FLNA) and myelin proteolipid protein (PLP1)) from Table 2 for validation on the pooled lysates that were used for the iTRAQ experiment. Glyceraldehyde-3-phosphate dehydrogenase (GAPDH) were used as the loading control. As shown in Fig. 3a-b, the abundance of SYNPO was lower whereas GSTP1 and PLP1 level were higher in Lewy body dementias and its clinical subtypes. VIM was down-regulated in PDD. The level of NCAM did not show any variation among different groups. No clear band could be detected for COL6A3 and FLNA during the immunoblotting experiment. FLNA and COL6A3 are high molecular weight proteins and were identified with very low sequence coverage (FLNA, 3.2% and COL6A3, 1.4%; Table 2) in the iTRAQ experiment indicating the presence of miniscule levels of these proteins in the original samples. This could have attributed to the poor signals during the immunoblot experiment. Overall, we observed a consistent trend of regulation for at least four proteins (i.e. GSTP1, PLP1, VIM and SYNPO) in between the iTRAQ and immunoblot experiment of pooled lysates.

#### Immunoblotting of selected protein candidates on individual subjects

Next, we used the samples of all sixty-one subjects to probe the levels of the validated candidates by immunoblotting. Notably, *p*-value from the iTRAQ and immunoblot experiment of the pooled samples provided an estimate of the technical variation while *p*-value for this experiment will provide an estimate of the biological variation. This is crucial for the general applicability of the findings. The abundance of SYNPO was significantly lower in both PDD (*p* = 0.030) and DLB (*p* = 0.024) groups when compared with the control subjects (Fig. 3c-d). Further, the level of SYNPO did not differ significantly in between DLB and PDD group. On the other hand, VIM, PLP1 and GSTP1 failed to display significant alteration (*p* > 0.05) between control and either subtypes of Lewy body dementias. This indicates that wide biological variation within a group probably due to wider intra-group distribution of Braak stages can obscure the trends obtained from the immunoblot experiment of pooled lysates. Despite the negative trends for these three proteins, the data has important ramifications. First, these candidate proteins can be preferentially selected for a hypothesis-driven study in a different cohort of Lewy body dementia subjects. They may have higher chance to show significant difference in demented subjects compared to a randomly selected protein. Second, if a similar discovery driven study is planned with a similar experimental design and analysis criteria, these proteins could be the candidate of choice for a meta-analysis.

#### Correlation analyses

Correlation analyses were performed between the levels of selected candidate proteins in individual subjects and various neuropathological variables such as Braak Stage, CERAD, NP, NFT and LB scores, as well as Aβ42 concentrations. SYNPO levels was negatively correlated with CERAD (*ρ* = −0.34), NP (*ρ* = −0.43), LB (*ρ* = −0.33) and Aβ42 (*ρ* = −0.38). Even within the demented subjects, the correlation remained significant between SYNPO and NP (*ρ* = −0.38). In contrast, no significant correlation was observed between the other candidate proteins and neuropathological variables, except weak correlations were observed with CERAD (VIM, *ρ* = −0.27), Aβ42 (VIM, *ρ* = −0.26) and Braak stages (GSTP1, *ρ* = 0.27) among all subjects, but not when limiting to the demented subjects only. Detailed results of the correlation analyses are listed in Additional file 2: Table S2.

## Discussion

Previous reports are conflicting on whether to classify PDD and DLB as two clinical subtypes of Lewy body dementias or consider them as a part of a continuum of a heterogeneous, single disorder. Since the clinically adopted ‘one-year rule’ assumes that PDD and DLB have overlapping disease processes which are distinguished partly by quantitative differences in the amyloid plaque load [11, 16, 17], we believed that a proteome-wide comparison of DLB and PDD subjects that were matched by the tissue concentration of total Aβ42 and amyloid plaque load could settle this ongoing debate. The frontal cortex is critically involved in the cognitive processes and have been found by us and others to be subject to Lewy body pathology and neurochemical deficits [27, 31]. Santpere et al. found network level disruption of gene regulation including those involved in energy metabolism, protein folding and synaptic function [32]. Therefore, we selected a defined region of the prefrontal cortex (i.e. BA9) as a promising target for our proteomic analyses.

### Lack of substantial differences in proteomic signature between DLB and PDD

Our proteomic study consisted of an imprint from the BA9 area of neocortex containing 1914 proteins (FDR = 0%) from a surfactant-soluble fraction. In contrast, a previous iTRAQ-based study that used a surfactant-insoluble fraction isolated from the temporal lobe identified 106 proteins [24]. In our study, microtubule-associated protein 2, COL6A3, and FLNA were down-regulated only in PDD without showing any change in DLB samples. ‘Dementias’ group gave consistent pattern of response in most occasions thus cross-verifying the technical reliability of the iTRAQ experiment. In general, although PDD appeared to elicit wider perturbation of brain proteome compared to DLB based on the iTRAQ dataset and immunoblotting of pooled samples (Fig. 3a-b), the direction of deregulation was largely similar between these two subtypes of Lewy body dementias. Intriguingly, not a single protein from the shortlist (Table 2) showed opposite trend of regulation between PDD and DLB that is beyond the specified threshold of 1.3-fold. Unsurprisingly, the two-step immunoblot experiments involving pooled and individual subjects, failed to identify any protein that varies significantly between DLB and PDD (Fig. 3). This indicates that the clinically defined differences may not be driven by alterations in crude abundances of total protein levels in between DLB and PDD. This provides additional justification as to why studies using traditional approaches (e.g. ELISA) and targeting either CSF or blood failed to validate a single circulatory protein biomarker that can distinguish between DLB or PDD [33].

### Differences between DLB and PDD is likely driven by different burdens of concomitant Alzheimer’s pathology

The next question we faced was why no wide differences were observed in total protein-levels despite scanning almost 10% of human proteome. Whilst much of the available literature have shown an overlapping neuropsychology and neuropathology between DLB and PDD without clear-cut neurochemical differences between the two subtypes of Lewy body dementias, we were nevertheless able to gather a few studies that have reported differences between DLB and PDD using a multitude of approaches ranging from neuropathological, neurochemical to neuroimaging avenues [34, 35]. For example, using a neuroimaging approach that involves the use of [^11^C] Pittsburgh Compound B (PIB)-PET, an earlier and more extensive cortical Aβ deposition was shown in DLB compared to PDD [26]. Intriguingly, the reported differences in all the above-mentioned studies have been attributed to an underlying Aβ pathology. It is well known that apart from α-synuclein positive LBs and Lewy neurites, Alzheimer’s pathology co-exists in both DLB and PDD in the form of amyloid plaques as well as neuritic elements [35]. Aβ42, the 42-amino acid peptide derived from the amyloid precursor protein, is the main constituent of amyloid plaques. Accordingly, we measured the levels of total Aβ42 for the complete cohort of Lewy body dementia patients to estimate the relative involvement of Aβ pathology between DLB and PDD groups. We detected significantly higher levels of Aβ42 in DLB when compared to PDD or control group (Fig. 1a) which corroborates earlier findings showing higher Aβ burden in various brain regions of DLB patients [25–27]. As the total Aβ42 fractions include fibrillar Aβ42 which constitutes amyloid plaques [36], measurements of total Aβ42 is likely to provide a good estimate of amyloid plaque burden. Given that the matching of DLB and PDD subjects by total Aβ42 burden resulted in no candidate protein which could differentiate DLB from PDD in the proteomic dataset, it is therefore likely that the bulk of proteomic differences between DLB and PDD are driven by a concomitant Alzheimer’s pathology. This finding is consistent with recent studies where major overlap of CSF Aβ42 profile has been found between DLB and AD patients [37] and an aberrant CSF Aβ42 level predicted cognitive decline in DLB and PD patients [38, 39]. Alternatively, it may suggest the involvement of abnormal post-translational modifications or protein-protein interactions, defective intracellular trafficking or structural anomalies such as aberrant protein folding in the pathogenesis of Lewy body dementias.

### Unchanged expression of α-synuclein (SNCA)

SNCA, being the major constituent of LBs and Lewy neurites, is the focus of several genetic and biomarker studies in Lewy body dementias that failed to provide any consistent trend [35]. In another iTRAQ-based study, SNCA was identified as a surfactant insoluble protein in the frontal cortex where only one peptide was detected for SNCA, isoform 2–4 [24]. We identified at least six peptides for SNCA from the surfactant soluble fraction with a sequence coverage of 65.7%. We did not observe any alteration in the levels of SNCA in between the subtypes of dementia or control subjects in the iTRAQ dataset. This agrees with the above study where SNCA showed comparable levels between Parkinsonism-dementia complex and DLB when triton X-100 insoluble fractions from neocortex was tested by ELISA [24]. However, the higher LB loads in DLB compared to PDD observed in our cohort (Fig. 2d) suggests that other species of SNCA, e.g., those phosphorylated at Serine129, may be more directly implicated in LB pathogenesis [40] and should be further investigated. Importantly, none of the candidate proteins correlated with LB scores within the Lewy body dementias group (Additional file 2: Table S2), suggesting that differences in LB loads between DLB and PDD do not underlie their proteomic differences.

### Decrease in SYNPO level is indicative of a synaptic decline in Lewy body dementias

Dysfunctional Synaptic transmission is a known contributing factor for various types of dementia such as AD and VaD. Hence, we searched the dataset for various synaptic markers that were confidently identified in this cohort of subjects. Notable candidates were Synaptophysin, Synaptosomal-associated protein 25, Rab3A and SYNPO (Table 2). However, except SYNPO, none of them showed deregulation. SYNPO is enriched in post-synaptic dendritic spines of mature neurons and used as a marker of dendritic spines. It is a cytoskeletal protein that directly binds with F-actin like FLNA and may regulate spine morphology, motility and activity-dependent spine plasticity [41, 42]. To our knowledge, a couple of studies have examined the role of SYNPO in various forms of dementia. SYNPO was significantly decreased in frontal and parietal cortices of both early and definitive AD patients compared to age-matched control subjects [43]. Another study noted reduced density of SYNPO-labeled spine puncta only in AD cases with dementia, while SYNPO-labeled dendritic spines were maintained in pathologically confirmed AD with normal cognition [44]. Hence, the reduction of SYNPO probably indicates a declining synaptic function in the prefrontal cortex which may contribute to dementia. Perhaps, as SYNPO is detectable in plasma exosome and it shows lower level in patients with frontotemporal dementia and AD [45], a similar trend could be obtained in subjects of Lewy body dementias thus making it a potential biomarker candidate. Overall, considering that a synaptic protein presents a clear difference in its abundance, it will be worthwhile to target a synaptosomal preparation from autopsied tissues for similar studies in the future.

It is important to note the limitations of the study. First, a postmortem study captures a terminal snapshot of the proteome. It is possible that the proteomic landscape is distinct between DLB and PDD during the early stages of the disorders that converge into a set of common molecular pathways at an advanced stage making both subtypes virtually indistinguishable in terms of proteomic signature. However, it is difficult to collect matched brain samples at various time points during the temporal evolution of the disorder. Prospective studies through neuroimaging or systems biological approaches involving CSF specimens sampled at multiple time-points are important follow-up studies to the current observations. Second, it should be kept in mind that despite having targeted the whole proteome, we have analyzed the SDS soluble fraction of the brain proteome only. Plaques and aggregates such as LBs, amyloid deposits are poorly soluble in strong ionic detergents and can get lost during protein extraction even when 2% SDS is used. This could offset the significantly high difference of LB score between control and diseased groups resulting in false negatives. To check this, we compared the protein recovery (%weight of starting tissue) among the three groups (control, DLB and PDD). We assume that if the aggregates (amyloid plaques or LBs) are lost during protein extraction, the protein yield should be considerably low for DLB and PDD groups compared to control as there is higher proportion of aggregates in the diseased subjects. Further, proteins integral to LBs and amyloid deposits should show an artefactual down-regulation in DLB or PDD group due to incomplete sampling. However, no significant decrease was observed in between the mean protein recovery of any two groups (Additional file 2: Figure S2). In addition, SNCA and Amyloid beta A4 protein, which are the main component of LBs and amyloid aggregates respectively, had been confidently detected by the iTRAQ experiment without showing any deregulation. Further SNCA was one of the top 15 proteins in the dataset when sorted in terms of sequence coverage. Overall, non-uniform extraction with 2% SDS is unlikely to change the major conclusions of this study. Third, as Braak stages are linked to plaques and tangle burden, incomplete extraction of proteins with increasing Braak stages could lead to artifacts during group-wise comparison of proteomic landscape. To check the possible extent of confounding related to non-uniform distribution of Braak stages among different groups, we analyzed the relationship of protein yield with Braak stages (*n* = 55) using Spearman rank (ρ) correlation. This was followed by one-way ANOVA where mean protein recovery of all subjects, stratified into three categories of Braak stages (i.e. 0-II, III-IV, V-VI) were compared. None of them revealed any significant difference (Additional file 2: Figure S3) indicating that our extraction protocol worked uniformly irrespective of the Braak stages of the subjects. Finally, iTRAQ experiments are well-known to distort the protein ratios towards unity [46]. That may also contribute to false negatives. Keeping that in mind, we have used the lowest possible cut-off (i.e. 1.3-fold) in this study and manually incorporated additional candidates during short-listing and immunoblot validation. However, the methodology and MS instrument used for this study have been successfully utilized in the past on clinical brain samples to detect low levels of fold changes [18, 19]. Hence, ‘ratio distortion’ is unlikely to affect the major findings of this study.

In conclusion, we report one of the largest detergent-soluble proteomic dataset pertaining to Lewy body dementias using an iTRAQ-2D-LC-MS/MS based clinical proteomic approach. In this system-wide neuroproteomic study comparing a well characterized cohort of PDD and DLB patients pre-selected for comparable total Aβ42 and amyloid plaque burden, our results indicated a remarkable lack of differences in total protein levels in BA9 area of prefrontal cortex between DLB and PDD. This suggests that PDD and DLB are likely part of the same spectrum of Lewy body dementias rather than distinct entities, and the neurochemical changes noted by us and others [12–15] may be associated with differential burden of concomitant AD-related pathology or may be linked to changes in post-translational modifications or protein-protein interactions, defects in intracellular trafficking or structural anomalies such as aberrant protein folding. We also report SYNPO as a potential candidate biomarker for Lewy Body Dementias. Our study has implications in the therapeutic approaches for Lewy body dementias, as amyloid-targeting therapeutic strategies may be predicted to show different efficacies in DLB versus PDD.

## Methods

### Reagents

Unless indicated, all reagents and assay kits were purchased from Sigma-Aldrich (St. Louis, MO, USA).

### Patients and clinical assessments

Postmortem tissues from the prefrontal cortex (Brodmann area BA9) were obtained from PDD and DLB patients, along with aged non-demented controls from Stavanger University Hospital, Newcastle University and the London Neurodegenerative Diseases Brain Bank, the UK centers being part of the Brains for Dementia Research network (http://www.brainsfordementiaresearch.org.uk/). Informed consent was obtained from subjects or their next-of-kin prior to the removal of brains, and the study has Institutional Review Board (IRB) approvals from the UK (National Research Ethics Service 08/H10104) and Singapore (National University of Singapore IRB 12-062E). Neuropathologic assessments were performed for all subjects, and included Braak staging of neurofibrillary changes, CERAD scoring criteria, Newcastle/McKeith Criteria for Lewy body disease, and National Institute on Aging—Alzheimer’s Association guidelines. [2, 47–49] In addition, semi-quantitative scores of NP, NFT and LB using a four point scale (0, none; 1, sparse; 2, moderate; 3, severe) were assessed as previously described [27]. Controls were neurologically normal and did not have any history of dementia or psychiatric disease. For the patients, final differential diagnoses between PDD and DLB were based on clinicopathological consensus diagnosis considering the ‘one-year rule’ [2].

### Aβ42 Elisa

To measure total Aβ42 levels which has been shown to contain the highly aggregated fibrils of Aβ42 found in neuritic plaques [36], chunks of pre-frontal cortex (BA9) were homogenized in 5 M guanidine/50 mM Tris-HCl buffer as previously described [50]. The Aβ42 was measured using a sandwich ELISA (Invitrogen, Carlsbad, CA, USA, expressed in ng/mg protein) following the manufacturer’s protocol.

### Sample preparation for proteomics and immunoblotting

Frozen tissue samples from BA9 area were processed as described earlier with minor modifications [18]. Briefly, tissue samples were homogenized in lysis buffer [2% sodium dodecyl sulfate (SDS); 20 mM di-thiothreitol (DTT), 100 mM Tris, pH 7.9, with protease inhibitor cocktail (cOmplete™, Roche, Mannheim, Germany) and 2 mg/ml Pepstatin A and PhosSTOP (Roche)] using a Bullet Blender (Next Advance, New York, NY, USA; speed 8) for 10 min. The crude extracts were then centrifuged at 20600 g for 15 min. The supernatant was mixed with 4 volumes of ice cold 100% acetone by vortexing and kept at −20 °C overnight to precipitate the proteins. The lysates were centrifuged at 15000 g for 10 min to collect the protein pellet. The pellets were washed with 90% acetone to remove remaining contaminants and collected by centrifugation at 15000 g for 10 min. The acetone-precipitated proteins were re-dissolved in lyses buffer (6% SDS, 20 mM DTT, 100 mM Tris, pH 7.9) and stored in aliquots at −80 °C for long-term use. The samples were processed at 4 °C. The protein concentration was determined by 2D Quant kit (Amersham Biosciences, Piscataway, NJ, USA).

### Discovery proteomics

The in-gel digestion, peptide labeling, chromatographic separation and mass spectrometric analysis were performed following previously established methods [30, 51] with minor modifications.

### In-gel tryptic digestion and isobaric labeling

The samples (250 μg of protein/group) were subjected to a denaturing PAGE using a 4% - 6% - 25% gel following a similar procedure as described previously [30]. Briefly, proteins were migrated through the 4% into the 6% layer, but were retarded by the 25% layer, thus concentrating them in a narrow strip at the end of the stacking gel. The diced gel bands were then reduced [5 mM tris-(2-carboxyethyl) phosphine, 60 °C, 1 h] and alkylated (10 mM methyl methanethiosulfonate in isopropanol, room temperature, 45 min in the dark) before being digested with 12.5 ng/μL of sequencing-grade modified trypsin (Promega, Madison, WI, USA) in 50 mM triethylammonium bicarbonate buffer (TEAB), 2% acetonitrile (ACN) for overnight at 37 °C. The peptides were extracted with 50% ACN, 5% acetic acid and vacuum-centrifuged to dryness. The dried peptides were reconstituted into 0.5 M TEAB and labeled with respective isobaric tags of 8-plex iTRAQ Reagent Multi-Plex kit (Applied Biosystems, Foster City, CA, USA). The labeling scheme was as follows; control (label: 113, 116), DLB (label: 114, 117), PDD (label: 115, 118), Dementias, i.e. combined samples of all DLB and PDD subjects (label: 119) and pooled groups (label: 121) where all samples across groups were combined. The labeled samples were then combined and dried using vacuum-centrifugation.

### Electrostatic repulsion and hydrophilic interaction chromatography (ERLIC)

The mixture of iTRAQ-labeled peptides was fractionated using a PolyWAX LP anion-exchange column (4.6 × 200 mm, 5 μm, 100 Å) (PolyLC, Columbia, MD, USA) on a Prominence UFLC system (Shimadzu, Kyoto, Japan) and monitored at 214 nm. Fifty five fractions were collected during a 65 min gradient of 100% buffer A (0.1% formic acid (FA), and 10 mM ammonium formate in 85% ACN) for 5 min, 0–25% of buffer B (0.1% FA in 30% ACN) for 35 min, 25–100% of buffer B for 10 min, 100% of buffer B for 10 min, and 100% of buffer A for last 5 min at a flow rate of 1 ml/min [52]. Eluted fractions were pooled into 20 factions depending on the peak intensities. They were dried in a vacuum centrifuge and redissolved in 0.1% FA in 3% ACN for LC-MS/MS analysis.

### Reverse phase LC-MS/MS analysis using QSTAR

Each fraction of redissolved iTRAQ-labeled peptides was sequentially injected in triplicate and separated in a home-packed nanobore C18 column (75 μm ID × 15 cm, 2.4 μm particles, Reprosil-Pur C18-AQ, Dr. Maisch GmbH, Ammerbuch, Germany) with a picofrit nanospray tip (New Objectives, Woburn, MA, USA) on a Tempo nano-MDLC system coupled with a QSTAR Elite Hybrid MS (Applied Biosystems/MDS-SCIEX). Each fraction was independently analyzed by the LC-MS/MS over a gradient of 90 min with the constant flow rate of 300 nl/min [29, 30]. Data acquisition in QSTAR Elite was set to positive ion mode using Analyst QS 2.0 software (Applied Biosystems, Foster City, CA, USA). The precursors with a mass range of 300–1600 m/z and calculated charge from +2 to +5 were selected for fragmentation. For each MS spectrum, 5 most abundant peptides at most above a 10-count threshold were selected for MS/MS, and the selected precursor was dynamically excluded for 15 s with a mass tolerance of 50 mDa. Smart information-dependent acquisition was activated with automatic collision energy and automatic MS/MS accumulation. The fragment intensity multiplier was set to 20 and maximum accumulation time was 2 s.

### Mass spectrometric raw data analysis

Spectra acquired from each of the technical and experimental replicates were submitted alone and together to ProteinPilot Software (v 3.0, Revision Number: 114,732, Applied Biosystems) for peak list generation, protein identification and quantification against the concatenated target-decoy Uniport Human database (v 120,312). User defined parameters in ProteinPilot software were configured as described previously [51] with minor modifications as follows: (ii) Specify Processing, Quantitate and Bias Correction. Default precursor and MS/MS tolerance for QSTAR ESI-MS instrument were adopted automatically by the software. The FDR of both peptide and protein identification were set to be less than 1% (FDR = 2.0*decoy_hits/total_hits).

### Immunoblotting

Immunoblotting was performed after 10% or 12% SDS-PAGE by probing with primary antibodies at the indicated dilutions: anti-GAPDH (1: 1000, mouse monoclonal; Milipore, Billerica, MA, USA), anti-COL6A3 (1:200, mouse monoclonal; Santa Cruz Biotech, Santa Cruz, CA, USA), anti-GSTP1 (1:1000, rabbit polyclonal; Abcam, Cambridge, UK), anti-FLNA (1:1000; rabbit polyclonal; Cell Signaling Technology, Danvers, MA, USA), anti-NCAM (1:10,000, rabbit polyclonal; Santa Cruz Biotech), anti-PLP1 (1:1000, rabbit polyclonal; Abcam), anti-SYNPO (1:500, rabbit polyclonal; Abcam), anti-VIM (1:1000, rabbit polyclonal; Genscript, Piscataway, NJ, USA). Twenty to fifty micrograms of proteins were used depending on the sensitivity of the specific antibody. Immunoreactivity was detected by using an HRP chemiluminescent substrate reagent kit (Invitrogen, Carlsbad, CA). A pooled sample was used to normalize the inter-gel variation between repeated runs for the same protein. Immunoreactivities of antibodies were visualized with Luminata™ Forte or Crescendo Western HRP substrate (Merck Millipore, Germany) and quantified with the Alliance 4.7 image analyser (UVItec, UK).

### Statistical analyses

Statistical analyses were performed using SPSS software version 21 (IBM, Armonk, NY, USA) and GraphPad Prism (San Diego, CA, USA). Data were first tested for normality using Komogorov-Smirnov test when deciding the use of parametric vs. non-parametric tests. Normally distributed data were compared using one-way ANOVA followed by *post-hoc* Bonferroni tests, while ordinal or non-normally distributed data (i.e., Aβ42 level, CERAD score) were compared using Kruskal-Wallis ANOVA followed by *post-hoc* Dunn’s tests. Similarly, independent-samples *t*-tests or Mann-Whitney U tests were used to compare means for two groups of cases, while correlations between variables were analyzed with either Pearson’s product moment correlation or with Spearman’s rank correlations. Statistical significance is accepted at *p* < 0.05.

## Additional files

Additional file 1: Table S1. Complete information of the full list of the qualified proteins (Unused prot score > 2) obtained from the bias corrected iTRAQ data set. (XLSX 555 kb)

Additional file 2: Figure S1. Frequency distribution of fold variation between the experimental replicates. **Figure S2.** Interleaved scatter plot showing the protein yield (% tissue weight) across three groups (control, DLB and PDD). **Figure S3.** Interleaved scatter plot showing the changes in protein yield (% tissue weight) with increasing Braak stages. **Table S2.** Correlation between selected candidates’ protein levels and neuropathological variables of the subjects included in the study. (DOCX 268 kb)

## Abbreviations

2D–LC-MS/MS: two dimensional-liquid chromatography-tandem mass spectrometry
ACN: acetonitrile
AD: Alzheimer’s disease
Aβ: β-amyloid
BSA: bovine saline albumin
CERAD: Consortium to Establish a Registry for Alzheimer’s Disease
COL6A3: Collagen Type VI alpha 3 chain
CSF: cerebrospinal fluid
DLB: dementia with Lewy bodies
DTT: dithiothreitol
ERLIC: electrostatic repulsion and hydrophilic interaction chromatography
FA: formic acid
FDR: false discovery rate
FLNA: filamin A, alpha
GAPDH: glyceraldehyde-3-phosphate dehydrogenase
GSTP1: glutathione-S-transferase P
HRP: horseradish peroxidase
iTRAQ: isobaric tag for relative and absolute quantitation
LBs: Lewy bodies
NCAM1: neural cell adhesion molecule 1
NFT: neurofibrillary tangles
NP: neuritic plaque
PD: Parkinson’s disease
PDD: Parkinson’s disease dementia
PLP1: myelin proteolipid protein
SDS: sodium dodecyl sulfate
SNCA: α-synuclein
SOD1: superoxide dismutase 1
SYNPO: synaptopodin
TEAB: triethylammonium bicarbonate
VaD: vascular dementia
VIM: vimentin
