# Supplementary material for: An iTRAQ-based proteomic analysis reveals dysregulation of neocortical synaptopodin in Lewy body dementias
Source: Mol Brain. 2017 Aug 11;10:36. doi: 10.1186/s13041-017-0316-9 (PMC5553757; doi:10.1186/s13041-017-0316-9)
Supplement: Supplementary file 2 — Frequency distribution of fold variation between the experimental replicates. Figure S2. Interleaved scatter plot showing the protein yield (% tissue weight) across three groups (control, DLB and PDD). Figure S3. Interleaved scatter plot showing the changes in protein yield (% tissue weight) with increasing Braak stages. Table S2. Correlation between selected candidates’ protein levels and neuropathological variables of the subjects included in the study. (DOCX 268 kb) [file 13041_2017_316_MOESM2_ESM.docx]

Datta *et al.* An iTRAQ-based proteomic analysis reveals dysregulation of neocortical synaptopodin in Lewy body Dementias

**Figure S1**


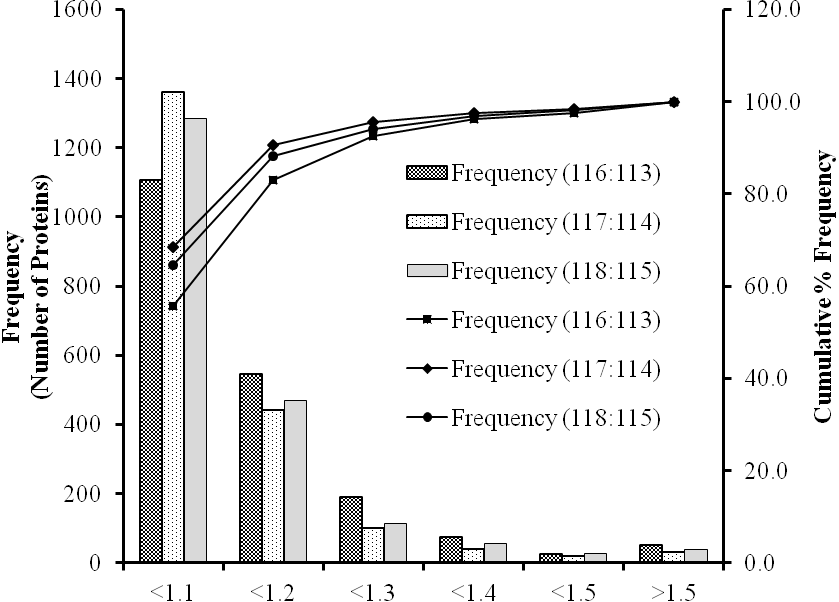


**Figure S1.** Frequency distribution of fold variation between the experimental replicates. Each group was labeled in duplicate (control: 113, 116; DLB: 114, 117; PDD: 115, 118). The fold deviation from 1 between experimental replicates (113/116, 114/117 and 115/118) were used to draw the frequency histogram. The primary vertical axis represents the corresponding number of the proteins (bars) having different fold variation that was plotted in the horizontal axis. The secondary vertical axis represents the cumulative % of the counted proteins (lines) where 100% equals to 1988 proteins. About 94.7% (average) of the proteins had less than 1.3-fold variation. Accordingly, regulation cut-off was set at 1.3-fold.

**Figure S2**

**Figure S2:** Interleaved scatter plot showing the protein yield (% tissue weight) across three groups (control, DLB and PDD). The Y-axis is showing the mean and individual protein recovery following protein extraction with a 2% SDS-containing buffer. Average recovery of proteins did not vary significantly (one-way ANOVA followed by post-hoc Bonferroni’s multiple comparison tests) among the groups. Data was presented as mean ± SD. SD, standard deviation.

**Figure S3**

**Figure S3:** Interleaved scatter plot showing the changes in protein yield (% tissue weight) with increasing Braak stages. The Y-axis is showing the mean and individual protein recovery following protein extraction with a 2% SDS-containing buffer. The X-axis represents all subjects (n = 55) with various Braak stages stratified into three categories (0-II, III-IV and V-VI). No significant difference was observed (one-way ANOVA followed by post-hoc Bonferroni’s multiple comparison tests) in between the mean yield of proteins for any two groups. Data was presented as mean ± SD. SD, standard deviation

Datta *et al.* An iTRAQ-based proteomic analysis reveals dysregulation of neocortical synaptopodin in Lewy body Dementias

**Table S1**

Complete information of the full list of the qualified proteins (Unused prot score > 2) obtained from the bias corrected iTRAQ dataset.

**Available as EXCEL file**

**Table S2**

Correlation between selected candidates’ protein levels and neuropathological variables of the subjects included in the study.

|  | Aβ42^a^,  *ρ* (p-value) | Braak stage^b^,  *ρ* (p-value) | CERAD score,  *ρ* (p-value) | NP score,  *ρ* (p-value) | NFT score,  *ρ* (p-value) | LB score,  *ρ* (p-value) |
| --- | --- | --- | --- | --- | --- | --- |
| SYNPO levels |  |  |  |  |  |  |
| All | **-0.381 (0.002)** | -0.105 (0.443) | **-0.336 (0.014)** | **-0.425 (0.001)** | -0.168 (0.198) | **-0.327 (0.010)** |
| Dementia only | -0.159 (0.326) | 0.120 (0.459) | -0.124 (0.451) | **-0.383 (0.016)** | -0.105 (0.526) | -0.078 (0.632) |
| VIM levels |  |  |  |  |  |  |
| All | **-0.257 (0.046)** | 0.044 (0.750) | **-0.274 (0.047)** | -0.241 (0.064) | -0.067 (0.610) | -0.135 (0.300) |
| Dementia only | -0.227 (0.159) | 0.204 (0.207) | -0.242 (0.138) | -0.139 (0.398) | 0.153 (0.352) | 0.070 (0.667) |
| PLP1 levels |  |  |  |  |  |  |
| All | -0.016 (0.903) | 0.034 (0.807) | 0.103 (0.462) | -0.201 (0.124) | 0.030 (0.818) | -0.201 (0.121) |
| Dementia only | 0.060 (0.714) | 0.263 (0.101) | 0.193 (0.240) | -0.136 (0.408) | 0.062 (0.706) | -0.141 (0.387) |
| GSTP1 levels |  |  |  |  |  |  |
| All | -0.063 (0.629) | **0.273 (0.043)** | 0.165 (0.237) | -0.148 (0.260) | 0.190 (0.146) | 0.004 (0.976) |
| Dementia only | -0.001 (0.997) | 0.227 (0.160) | 0.216 (0.186) | -0.285 (0.078) | 0.174 (0.291) | -0.029 (0.858 |

Abbreviation: CERAD, Consortium to Establish a Registry for Alzheimer’s disease (see reference 26); GSTP1, glutathione S-transferase; LB, Lewy body; NFT, neurofibrillary tangle; NP, neuritic plaque; PLP1, myelin proteolipid protein; *ρ*, Spearman’s rho; SYNPO, synaptopodin; VIM, vimentin.

^a^Total Aβ42 concentrations are expressed in ng / mg brain protein.

^b^Braak stages are grouped into 0-II, III-IV and V-VI. Data not available for 6 controls.
